# Changes in sleep duration and 3-year risk of mild cognitive impairment in Chinese older adults

**DOI:** 10.18632/aging.102616

**Published:** 2020-01-03

**Authors:** Qi Zhu, Hui Fan, Xiaoning Zhang, Chao Ji, Yang Xia

**Affiliations:** 1 Department of Preventive Medicine, North Sichuan Medical College, Nanchong, China; 2 Institute of Basic Medicine, Shandong First Medical University and Shandong Academy of Medical Sciences, Shandong, China; 3 Department of Clinical Epidemiology, Shengjing Hospital of China Medical University, Shenyang, China

**Keywords:** sleep duration, mild cognitive impairment, aging, Chinese

## Abstract

Objective: This study aimed to determine whether changes in sleep duration are associated with a higher risk of mild cognitive impairment (MCI) in older adults.

Results: By the 3-year follow-up, 592 participants developed MCI. Compared with the individuals who had an unchanged sleep duration, the odds ratio (95% confidence interval) for MCI was 1.44 (1.08-1.91) for those whose sleep duration increased by ≥2 h after multivariate adjustments. Moreover, changing from a long to moderate, but not short, sleep duration was negatively associated with the incidence of MCI (odds ratio: 0.65; 95% confidence interval, 0.45-0.93).

Conclusions: These findings suggest that increased sleep duration is associated with a higher risk of MCI in the elderly. Furthermore, a moderate duration of sleep (6-9 h) could serve as a possible strategy for prevention of MCI.

Methods: This longitudinal study was conducted with a nationally representative sample of 5419 older Chinese adults (≥65 years) from the 2008 and 2011 waves of the Chinese Longitudinal Healthy Longevity Survey. Sleep duration was assessed by a self-administered questionnaire. MCI was defined according to the Mini-Mental State Examination. An adjusted logistic regression model was used to explore the associations between changes in sleep duration and MCI.

## INTRODUCTION

Mild cognitive impairment (MCI) has been defined as an intermediate stage between normal aging and dementia that is characterized by cognitive deficits [1]. People with MCI tend to have a significantly higher risk of dementia with progression rates between 8% and 15% per year [2–4], which not only is a major cause of disability and mortality among elderly individuals but also has a profoundly detrimental effect on the family and other caregivers. With the rapidly aging population, MCI was prevalent in recent years. The prevalence of MCI among the aging population (60 years of age and above) was 14.71% in China [5]. It is thus essential to identify potentially modifiable determinants of MCI to develop effective preventive interventions.

Older adults commonly experience sleep problems, and abnormal sleep durations have been recognized to impair performance on attention and executive control tasks [6]. Poor sleep health, both self-reported long and short sleep durations, has been shown to be associated with cognitive decline in older adults [7–9], although not consistently. Moreover, a meta-analysis revealed a U-shaped nonlinear association between sleep duration and the risk of MCI [10]. However, sleep is a dynamic process and sleep patterns change across the life course [11]. A one-time measurement of sleep duration is unable to determine whether observed associations between long or short sleep and MCI are generated by persistent exposure or whether decreased or increased sleep duration also confer risk. The majority of research has focused on the MCI risk of sleep duration as a single assessment at baseline. One prospective study among Japanese elderly individuals reported that increased sleep duration is associated with dementia [12]. However, few studies have focused on the relationship between changes in sleep duration and MCI, which represents early-stage dementia [13]. Another Whitehall II Study [14] found an increase from 7 or 8 h sleep and a decrease from 6 to 8 h of sleep were associated with lower cognitive function in the middle-aged individuals. Considering the high prevalence of MCI among the elderly, the associations between changes in sleep duration and the development of MCI in the elderly need to be further investigated.

Therefore, based on data from two waves of the Chinese Longitudinal Healthy Longevity Survey (CLHLS) occurring from 2008 to 2011, this study aimed to evaluate whether changes in sleep duration over a 3-year period were associated with the subsequent risk of MCI and to consider several potential confounding factors among older adults from China.

## RESULTS

### Characteristics of the study population

Of the 5419 participants at baseline, 596 had developed MCI at follow-up. The incidence of MCI was 11.0%. The mean (standard deviation) age of the participants was 79.8 (9.72) years old, ranging from 65 to 111, and 49.5% of them were male.

Baseline characteristics stratified by categories of changes in sleep duration are shown in Table 1. Compared with subjects with less or no changes in sleep duration, those reporting increases or decrease by ≥ 2h were older, were more likely to be illiterate, lived in a rural area and had activity of daily living (ADL) disability but were less likely to be married, were current smokers, had lower body mass index (BMI), had lower household income, had lower Mini-Mental State Examination (MMSE) scores and had poor self-reported sleep quality (all *P* <0.05). No significant difference was observed in sex, regular physical activity, alcohol drinking status, self-reported medical history or negative well-being.

**Table 1:** Baseline characteristics by changes in sleep duration ^a^.

	**Changes in sleep duration**					
	**decreased by ≥2 h (n = 1496)**	**decreased by 1 h (n = 726)**	**stable (n = 1055)**	**increased by 1 h (n = 701)**	**increased by ≥2 h (n = 1441)**	***P* value**
Age (years) ^b^	80.6 (80.1, 81.1)	78.0 (77.4, 78.7)	78.3 (77.7, 78.9)	78.4 (77.7, 79.1)	81.6 (81.1, 82.1)	<0.0001
Sex (males, %)	47.9	51.2	51.0	50.6	48.4	0.21
BMI (kg/m^2^)	21.1 (20.9, 21.3)	21.3 (21.0, 21.5)	21.4 (21.1, 21.6)	21.1 (20.9, 21.4)	20.7 (20.5, 20.9)	<0.0001
Residence (rural, %)	73.2	64.7	65.6	68.6	70.7	<0.01
Education (illiterate, %)	55.9	47.5	45.0	49.0	54.8	<0.0001
Marital status (married, %)	48.2	56.6	58.1	54.4	46.8	<0.0001
Household income (≥10000 Yuan, %)	54.0	56.8	60.4	61.2	52.9	<0.01
Regular physical Activity (%)	32.0	40.8	39.6	42.5	36.2	0.19
Smoking status (%)						
Smoker	22.0	25.3	24.5	22.3	21.6	0.04
Ex-smoker	16.1	15.2	15.8	14.7	15.3	0.70
Non-smoker	61.9	59.5	59.7	63.0	63.1	0.04
Drinker status (%)						
Drinker	20.4	22.0	22.2	20.4	21.4	0.56
Ex-drinker	14.9	11.7	12.6	13.0	13.1	0.64
Non-drinker	64.7	66.3	65.2	66.6	65.5	0.86
History of diseases (%)						
Hypertension	18.8	20.6	19.8	20.4	19.3	0.71
Diabetes	2.37	3.06	3.72	2.89	3.35	0.71
CVD	4.41	4.58	4.38	4.48	4.85	0.61
Sleep duration in 2008 (hours/per day)	9.11 (9.03, 9.20)	7.86 (7.73, 7.99)	7.76 (7.65, 7.86)	7.03 (6.90, 7.15)	6.43 (6.34, 6.52)	<0.0001
Sleep duration in 2011 (hours/per day)	5.85 (5.76, 5.94)	6.86 (6.73, 6.99)	7.75 (7.65, 7.86)	8.03 (7.89, 8.16)	9.74 (9.64, 9.83)	<0.0001
Sleep quality (%)						
Very good	16.9	16.4	14.0	16.0	12.2	0.11
Good	60.8	55.1	55.7	49.6	40.9	<0.0001
Fair	17.4	20.1	22.5	22.4	30.9	<0.0001
Bad	4.55	7.99	7.38	11.1	14.4	<0.0001
Very bad	0.35	0.41	0.42	0.90	1.60	<0.01
ADL disability (%)	3.54	3.99	2.56	2.43	4.86	0.02
Negative well-being	9.39 (9.31, 9.46)	9.45 (9.35, 9.56)	9.41 (9.33, 9.50)	9.44 (9.33, 9.55)	9.33 (9.26, 9.41)	0.29
MMSE scores in 2008	26.9 (26.8, 27.1)	27.5 (27.3, 27.7)	27.5 (27.3, 27.7)	27.5 (27.3, 27.7)	27.0 (26.8, 27.1)	<0.0001
MMSE scores in 2011	25.3 (25.0, 25.6)	26.2 (25.8, 26.7)	26.3 (25.9, 26.6)	26.0 (25.6, 26.5)	24.6 (24.3, 24.9)	<0.0001

Abbreviations: BMI, body mass index; ADL, Activities of daily living; CVD, cardiovascular disease; MMSE, Mini-Mental State Examination.

^a^ Analysis of variance or logistic regression analysis.

^b^ Mean (95% confidence interval) (all such values).

### Associations of changes in sleep duration with incidence of MCI

The analyses of changes in sleep duration as a continuous variable showed a significant effect (Odds ratio (OR) = 1.09; 95% confidence interval (CI) = 1.05-1.13). Table 2 presents the crude and adjusted associations between changes in sleep duration and risk of MCI when using changes in sleep duration as a categorical variable. In the final multivariate models, adjusted OR (95% CI) for incident MCI in subjects who increased their sleep duration by ≥2h compared with those whose sleep duration did not change was 1.44 (1.08-1.91). In contrast, risk did not differ between decreased sleep duration and stable sleepers (OR = 0.95; 95% CI = 0.72-1.28).

**Table 2:** Impact of change in sleep duration on the risk of mild cognitive impairment ^a^.

	**Changes in sleep duration**					**Continuous variable**
	**decreased by ≥2 h**	**decreased by 1 h**	**stable**	**increase by 1 h**	**increase by ≥2 h**	
No. of subjects	1496	726	1055	701	1441	
Incident MCI	169	58	95	59	215	
Model 1 ^c^	1.29 (0.99, 1.68) ^b^	0.88 (0.62, 1.23)	1.00 (reference)	0.93 (0.66, 1.30)	1.77 (1.38, 2.30)	
Model 2 ^d^	1.03 (0.78, 1.37)	0.91 (0.63, 1.30)	1.00 (reference)	0.92 (0.64, 1.31)	1.33 (1.02, 1.76)	
Model 3 ^e^	0.95 (0.72, 1.28)	0.91 (0.63, 1.30)	1.00 (reference)	0.96 (0.66, 1.37)	1.44 (1.08, 1.91)	

Abbreviations: MCI, mild cognitive impairment.

^a^Multiple logistic regression.

^b^Adjusted odds ratios (95% confidence interval) (all such values).

^c^Model 1 was crude model.

^d^Model 2 was adjusted for age, sex, BMI, smoking status, drinking status, residence, education, marital status, income, regular physical activity, self-reported medical history (hypertension, diabetes, cardiovascular disease).

^e^Model 3 was same as model 2 plus sleep quality, negative well-being, baseline sleep duration and baseline cognitive function (MMSE scores).

When we cross-classified participants according to sleep duration in 2008 and 2011, an increase in sleep duration from moderate to long was significantly associated with risk of MCI (OR, 1.46; 95% CI, 1.02-2.09). Similarly, there was a higher risk between an increase in sleep duration from short to long and MCI (OR, 2.18; 95% CI, 1.41-3.39) compared with persistent short sleepers. In contrast, decreased sleep duration from long to moderate was associated with a reduced risk of MCI (OR, 0.65; 95% CI, 0.45-0.93) (Table 3).

**Table 3:** Development of mild cognitive impairment by subgroups of sleep duration across 2008 and 2011^a^.

**Sleep duration in 2008**	**sleep duration in 2011**		
	**Moderate (6-9 h)**	**Short (≤ 6 h)**	**long (≥ 9 h)**
Moderate (6-9 h)			
No. of subjects	954	665	726
Incident MCI	64	67	103
Model 1 ^c^	1.00 (reference) ^b^	1.56 (1.09, 2.23)	2.30 (1.66, 3.21)
Model 2 ^d^	1.00 (reference)	1.26 (0.86, 1.85)	1.44 (1.01, 2.06)
Model 3 ^e^	1.00 (reference)	1.29 (0.88, 1.89)	1.46 (1.02, 2.09)
Short (≤ 6 h)			
No. of subjects	484	676	323
Incident MCI	32	50	62
Model 1	0.89 (0.56, 1.40)	1.00 (reference)	2.98 (2.00, 4.45)
Model 2	0.89 (0.55, 1.43)	1.00 (reference)	2.31 (1.51, 3.56)
Model 3	0.82 (0.50, 1.32)	1.00 (reference)	2.18 (1.41, 3.39)
Long (≥ 9 h)			
No. of subjects	582	375	634
Incident MCI	58	45	115
Model 1	0.50 (0.35, 0.70)	0.82 (0.50, 1.32)	1.00 (reference)
Model 2	0.65 (0.45, 0.94)	0.70 (0.47, 1.04)	1.00 (reference)
Model 3	0.65 (0.45, 0.93)	0.70 (0.46, 1.03)	1.00 (reference)

^a^ Multiple logistic regression.

^b^ Adjusted odds ratios (95% confidence interval) (all such values).

^c^ Model 1 was crude model.

^d^ Model 2 was adjusted for age, sex, BMI, smoking status, drinking status, residence, education, marital status, income, regular physical activity, self-reported medical history (hypertension, diabetes, cardiovascular disease).

^e^ Model 3 was same as model 2 plus sleep quality, negative well-being, baseline sleep duration and baseline cognitive function (MMSE scores).

## DISCUSSION

This large-scale population-based study examined the associations between changes in sleep duration and the development of MCI. The results of this study indicated that an increase in sleep duration was significantly associated with a higher risk of developing MCI among older Chinese adults. After adjustment for potential confounders, an extreme increase in sleep duration (≥ 2 h) had a 1.46-fold higher risk of MCI than individuals who had an unchanged sleep duration. Moreover, going from a long sleep duration of ≥ 9 h to a moderate sleep duration (6-9 h) was negatively associated with MCI relative to those who had an unchanged long sleep duration.

Little evidence has explored the associations between changes in sleep duration and the incidence of MCI. One recent prospective study among Japanese elderly participants found that an increase in sleep duration of ≥ 2h was related to a higher risk of dementia (hazard ratio, 1.72; 95% CI 1.33-2.22) [12]. Another Whitehall II study investigated the associations between changes in sleep duration and cognitive function and demonstrated that going from a regular pattern of 6-8 h per night to shorter and longer durations of sleep were associated with poorer cognitive function [14]. However, the absence of baseline cognitive function data meant that the associations observed in this study could be attributed to the possible reverse causality, participants with baseline cognitive impairment are more likely to develop adverse sleep patterns during the follow-up period. In our study, all subjects had repeated measurements of sleep-related factors and cognitive function. We analyzed the association between changes in sleep duration and the risk of MCI with participants having MCI at baseline being excluded, which would provide more valid findings.

Consistent with the aforementioned findings, this study suggested that subjects whose sleep duration increased by ≥ 2 h were at a higher risk of developing MCI. Although short sleep duration has been associated with worse cognitive function, memory impairment and psychomotor slowness [15–17], we did not find a significant association between a decrease in sleep duration and incident MCI. This may be partly because aging is associated with shorter sleep duration and may lead to age-specific “normal” values of sleep duration.

In addition, we extend previous studies by demonstrating how different patterns of sleep change affect the risk of incident MCI. Increased sleep duration in baseline moderate sleepers was associated with a higher risk of MCI compared with those remained moderate sleepers. An increase from baseline short sleep to long sleep durations was also associated with the development of MCI compared with persistent short sleep durations, which suggested that increased sleep duration is more deleterious than the maintenance of short sleep durations in older adults. Furthermore, going from a long sleep duration to a moderate sleep duration was associated with a lower risk of MCI compared with those with persistent long sleep durations, which means that reduced sleep duration in long sleepers has a beneficial effect on MCI risk. Collectively, these results indicate that an increase in sleep duration plays an important role in the progression of MCI; it may serve as an early biological marker of MCI. Moreover, sleep restriction in long sleepers would be useful in the prevention of MCI, and maintaining moderate sleep durations is optimal for cognitive function.

The exact mechanisms underlying the association between increased sleep duration and incident MCI has not yet been clarified. However, some potential explanations can be suggested. First, longer sleep durations have been associated with elevated levels of inflammatory markers [18–20], and low-grade inflammation promotes the development of cognitive impairment and dementia [21, 22]. Second, long sleepers have been reported to have more sleep disturbances [23], and previous studies have indicated that sleep disturbances are predictive of Alzheimer’s disease and other dementias [24]. Third, accumulation of amyloid β, a pathological hallmark of dementia, is enhanced during deranged sleep [25]. Longer durations in the recumbent position possibly increases the period with high intracranial pressure and may subsequently alter the cerebrospinal/interstitial fluid dynamics and compromise β-amyloid clearance during sleep [26], which has been implicated in the pathophysiology of cognitive dysfunction [27]. Fourth, previous studies have shown that homeostatic regulation of sleep duration interacts with circadian rhythm [28, 29]. Thus, a considerable increase in sleep duration may disrupt these circadian rhythms, thereby contributing to the development of MCI [30, 31]. Furthermore, prolonged sleep duration may also be indirectly related to cognitive impairment. For example, psychiatric disorders such as anxiety and depression are common in individuals with MCI [32] and are associated with sleep disturbances [33]. However, the associations between changes in sleep duration and MCI remained when the analysis controlled for sleep quality and negative well-being, an index that reflects the influence of depressive and anxiety symptoms.

The strengths of the present study include the use of a large representative sample of older adults and the adjustment of a considerable number of potential confounders, such as sociodemographic variables, socioeconomic status, lifestyle factors and history of diseases. However, there are several limitations to this study that should be mentioned. First, the examined sleep parameters relied on a single item that was self-reported; thus, this study was unable to differentiate nighttime sleep from time in bed or daytime napping. Nevertheless, previous studies have shown moderately good correlations between subjective estimates and objective measurements [34]. Furthermore, most large cohorts with repeatedly collected data on sleep duration remain reliant on self-reports, and it is important to recognize that self-reported sleep duration has been strongly associated with objectively ascertained health outcomes [35, 36]. This suggested our findings are likely to be valid. Second, although we adjusted for a considerable number of potentially confounding factors, MCI is affected by other sleep-related variables such as sleep disordered breathing, excessive daytime sleepiness, and circadian rhythm disturbances, which were not assessed in this study.

In conclusion, an increase in sleep duration was associated with a higher risk of MCI in an elderly population. Moreover, a moderate duration of sleep (6-9 h) could serve as a possible strategy for the prevention of MCI. Further studies are required to elucidate the exact mechanisms that underlie these associations.

## MATERIALS AND METHODS

### Study Population

The CLHLS is a large nationally representative survey designed to better understand the social, behavioral, environmental, and biological factors that affect human health and longevity. The participants were selected from 22 of 31 provinces in China in which 85% of the total Chinese population resided. The CLHLS was first administered in 1998, and the participants have been followed every two to three years. The questionnaires of the CLHLS collected data on basic information, life evaluation, personality, cognitive function, lifestyle, ADL, and personal background of the participants in each wave. A detailed description about the research design and data collection of the CLHLS has been published elsewhere [37].

The present study included individuals who had data in 2008 as a baseline and 2011 as a follow-up for data analysis (the latest datasets we could obtain when this study was carried out). A total number of 16954 participants were enrolled in 2008. In 2011, 5642 (33.3%) older adults died before the re-interview, and 2894 (17.0%) were lost to follow-up. Out of 8418 participants with follow-up, we further exclude subjects with missing cognitive or sleep data (n=1900), those self-reported diagnoses of dementia (n=37), those under 65 years old (n=283), those with incomplete information (n=343), and those suffered from MCI at baseline (n=436). As a result, data from 5419 subjects were analyzed. This study was approved by the Institutional Review Board of Duke University and Peking University, and all participants provided written informed consent.

### Assessment of MCI

Cognitive performance was measured by the Chinese version of MMSE [38], a widely used tool for the assessment of cognitive status in elderly subjects. It is a 14-question measure that tests five areas of cognitive function: orientation, registration, attention and calculation, recall, and language. The total score can ranging from 0 to 30. Previous studies have found that MMSE performance was highly influenced by education [39], and a considerable proportion of illiterate individuals (56.0%) were observed in this elderly population. Therefore, we adopted cutoff points based on education level, which is widely accepted and used in China [40], to define cognitive impairment: <18 for those without formal education, <21 for those with 1-6 years of education and <25 for those with more than six years of education.

### Assessment of sleep variables

Sleep duration was ascertained by the question “how long do you normally sleep?” According to the response, participants were categorized into the following three categories: short (≤ 6 h), moderate (6-9 h), and long (≥ 9 h) sleep durations. For the examination of changes in sleep duration from 2008 and 2011, the participants were divided into 5 groups: (1) decrease in sleep duration by ≥2 h, (2) decrease in sleep duration by 1 h, (3) stable sleep duration (changes in sleep duration by <1 h), (4) increase in sleep duration by 1 h, and (5) increase in sleep duration by ≥ 2 h. Sleep quality was assessed by the following question: “how do you rate your recent sleep quality?” Response categories included very good, good, fair, bad, and very bad.

### Assessment of other variables

Anthropometric parameters (height, weight, and waist circumference) were measured using a standard protocol. BMI was calculated as weight/height^2^ (kg/m^2^).

Additionally, age, sex, smoking status (never, former or current), drinking status (never, former or current), residence (rural or urban), education (illiterate or literate), marital status (married or unmarried) and household income (≥10000 Yuan or not) were obtained from a multidimensional questionnaire. Information on regular physical activity was collected using the question “Do you do exercise regularly at present, including jogging, playing ball, running and Qigong?” and recoded as yes or no. The ADL disability was measured by the Katz ADL Scale. The participants who could not independently perform at least one of the six activities (bathing, dressing, eating, toileting, indoor activities and continence) were defined as having ADL disability. A self-reported medical history (hypertension, diabetes, cardiovascular disease) was ascertained by two questions: (1) Are you suffering from this disease? (2) Have you been diagnosed by a physician? Only those who answered ‘yes’ to both questions were considered to have the corresponding disease. Although there is no direct measurement of depression, we used an index of negative well-being as a measure of depressive symptoms [41]. Negative well-being was measured by three items about neuroticism (I feel fearful and anxious), loneliness (I feel lonely or isolated) and perceived loss of self-worth (I feel useless with age). The subjects answered items on a five-point scale from “never” to “always”. We obtained scores by summing response codes, with higher scores indicating worse psychological well-being.

### Statistical analysis

Statistical analyses were performed using Statistical Analysis System 9.4 edition for Windows (SAS Institute Inc., Cary, NC, USA). Descriptive data are presented as the mean (95% CI) for continuous variables and as percentages for categorical variables. For baseline characteristic analyses, analysis of variance (ANOVA) was used to compare the differences in continuous variables between groups with different changes in sleep duration and logistic regression analysis for proportional variables. Multiple logistic regression analysis was constructed to estimate the ORs and 95% CIs for each change in sleep duration category associated with risk of MCI, with the stable sleep duration being the reference group. We categorized changes in sleep duration in this way to allow comparison with previous studies, but categorization reduced statistical power to detect associations. We therefore also fitted models with changes in sleep duration in hours as a continuous variable. For this analysis, three different models were fitted: Model 1 was a crude model without any adjustment; Model 2 adjusted for age, sex, BMI, education, marital status, residence, regular physical activity, income, smoking status, drinking status, and self-reported medical history at baseline; and Model 3 included variables in model 2 plus sleep quality, negative well-being, sleep duration and MMSE scores at baseline. In addition, further analyses were performed to examine whether the combination at two time points affected the risk of MCI. Therefore, nine subgroups were created according to combinations of the three sleep categories in 2008 and 2011. The multivariate logistic model was also used, with the reference group being the subgroup of participants who remained the same as at baseline. All *P*-values were two-sided, and *P* <0.05 was considered statistically significant.
